# Availability, affordability and costs of pediatric medicines in Mongolia

**DOI:** 10.1186/s12887-018-1123-x

**Published:** 2018-05-02

**Authors:** Gereltuya Dorj, Bruce Sunderland, Tsetsegmaa Sanjjav, Gantuya Dorj, Byambatsogt Gendenragchaa

**Affiliations:** 1 School of Pharmacy and Biomedicine, Mongolian National University of Medical Sciences, S.Zorig Street, Ulaanbaatar, 14210 Mongolia; 2 School of Pharmacy, Bentley, Curtin University, GPO Box U1987, Bentley, WA 6845 Australia; 3 School of Public Health, Mongolian National University of Medical Sciences, S. Zorig Street, Ulaanbaatar, 14210 Mongolia

**Keywords:** Essential medicines, Pediatric, Availability, Cost, Mongolia

## Abstract

**Background:**

The Essential Medicines List for Children (EMLc) was developed by the World Health Organization (WHO) to assist member countries to achieve Millennium Development Goals (MDG). The Government of Mongolia has adopted a National Essential Drug List (NEDL) with the seventh update published in 2014. The objective of this study was to determine the accessibility, availability and costs of essential pediatric medicines in Mongolia.

**Methods:**

A standardized methodology developed by the WHO and Health Action International (HAI) was employed to conduct a study on the availability, costs and affordability of pediatric medicines in Mongolia. A data collection tool collected information in regards to retail and wholesale availability and costs of essential pediatric medicines at pharmacy outlets during January and August of 2016.

**Results:**

Availability of individual essential pediatric medicines varied across the country. The average availability of medicines was 72.6% in the public sector (9.1–100%). Correspondingly, average availability of all selected medicines in the private sector was 76.7% (26.7–100%). Lowest price medicines were 2.45 times higher than the international reference price (IRP) in the private sector and was 1.95 times higher in the public sector. The lowest cost medicines in the public sector were more affordable for all conditions. The least affordable treatment was estimated to be for respiratory tract infections, or otitis media using amoxicillin clavulanic acid, suspension costing up to 1.03 days wages.

**Conclusion:**

Procurement, supply and distribution of essential pediatric medicines needs to be regularly investigated in order to identify the availability and costs of pediatric formulations in Mongolia.

## Background

Mongolia is a country with a vast land of 1.56 million km^2^ with a population of three million. The country is divided into 21 provinces (aimags) and a metropolitan city (Ulaanbaatar). Ulaanbaatar city has 9 districts and they are sub-divided into 152 khoroos. Most of the population lives in urban areas and approximately 20% is nomadic. Unemployment was about 10.6% and the inflation rate was 0.9% in 2016 [1]. According to the World Bank, Mongolia is a lower middle income country with an estimated Gross Domestic Product (GDP) of United States Dollar (USD) 11.76 billion in 2015. About 14% of the population lives on less than USD 1 per day [2]. The infant mortality rate was 15.3 per 1000 live births in 2014 [3].

### Pharmaceutical sector

The Drugs Act of Mongolia was approved in 1998 with an aim of ensuring good quality, effective and safe drugs [4].The Government subsequently promulgated the National Drug Policy of Mongolia (NDPM) in 2000 which was updated in 2014 [5]. Currently, 1496 pharmacies are operating in Mongolia of which 75% had one or two branches. There were 306 pharmacies working under the drug revolving fund (DRF) initiative [6].

The Essential Medicines List for Children (EMLc) [7] was developed by the World Health Organization (WHO) to assist member countries to achieve Millennium Development Goals(MDG) [8]. In December 2007, the WHO has also initiated the “Make Medicines Child Size” with a purpose of improving the accessibility of safe, effective and quality medicines for children by promoting awareness and action through research, regulatory measures and changes in government policy [9]. In accordance with WHO recommendations [10], the Government has adopted a National Essential Drug List since 1991 with regular updates, the seventh being completed in 2014 (7thEML). The NEDL contains 419 unique formulations, including 181 pediatric drugs [11]. There were 591 new drugs (salts or doses), 48 raw materials registered and the registration period was extended for a total of 428 drugs in 2014 [12].

In order to overcome barriers in supplying pediatric medicines and increase the accessibility to appropriate pediatric formulations, it is necessary to evaluate the availability and costs of essential medicines in pharmacy outlets and national medicines lists. Based on evidence and reliable scientific data, governments can apply necessary policy decisions and undertake interventions. Previous findings suggest that the availability and affordability of essential medicines needs to be improved in order to ensure accessibility to essential medicines in Mongolia [13]. However, no study has assessed the situation of essential pediatric medicines in Mongolia.

Therefore this study aimed to investigate the accessibility, availability and costs of essential pediatric medicines in Mongolia.

## Methods

A standardized methodology developed by the WHO and Health Action International (HAI) [14] was applied to conduct the study. The validation of the WHO/HAI methodology has been reported in several studies previously [15, 16]. A data collection tool was employed to collect availability information and retail and wholesale costs of essential pediatric medicines at pharmacy outlets during January and August of 2016. This timeline was selected due to the seasonal variables and high incidences of diseases in winter (January).

### Selection of pharmacy outlets

Data collection included six aimags/regions/provinces, Ulaanbaatar city, Erdenet city, Khuvsgul aimag, Dornod aimag, Govi-Altai aimag and Selenge aimag. The capital city Ulaanbaatar was selected as the major urban centre of Mongolia and an additional five districts were selected randomly from those which could be reached within a day’s travel from Ulaanbaatar city. The selection of study sites consistently included both urban and rural areas in all districts.

In each study area, five to ten pharmacy outlets were randomly selected from the list provided by the Health Development Centre, Ministry of Health and Sports of Mongolia.

The private sector sample included a total of 45 pharmacy outlets. Of those 11 private pharmacy outlets were located in public hospitals. Pharmacy outlets were selected based on active registration status and market volume size [17].

### Selection of medicines

As specified in the “Better Medicines for Children Project”, a core list of EMLc’s representative of commonly used medicines for treatment of various pediatric conditions prevalent in low middle/income countries was adopted for the study. The core list included pediatric dosage forms for 23 formulations plus one device [18].

The study included all formulations on the core- list but excluded Artemether+lumefantrine as it is used for treatment of malaria which is not prevalent in Mongolia. Alternative formulations were registered in Mongolia were added into the list of core medicines and were investigated. A total of 30 medicines and one device were surveyed in this study (Table 1).

**Table 1:** List of pediatric medicines surveyed in Mongolia

No.	Disease	Medicine	Formulation	Strength	Target size pack
1	Intestinal parasite	Albendazole	tablet	200 mg	2
2	Infectious disease	Amoxicillin	capsule	250 mg	10
	Infectious disease	Amoxicillin	suspension	125 mg	100 ml
3		Aminophyllin	injection	25 mg/ml	1 vial
4	Infectious disease	Amoxicillin_Clavulanic acid	tablet	125 mg + 125 mg, tab	10
	Infectious disease	Amoxicillin_Clavulanic acid	suspension	125 mg + 31.25 mg	100 ml
	Infectious disease	Amoxicillin_Clavulanic acid	suspension	250 mg + 62.50 mg	100 ml
5	Infectious disease	Azithromycin	capsule	250 mg	6
	Infectious disease	Azithromycin	suspension	200 mg/5 ml	100 ml
6	Asthma	Beclomethason	inhaler	50 mg/day	1 inhaler (200 doses)
7	Infectious disease	Benzylpenicillin	injection	600 mg = 1 million IU	1 vial
8	Seizure Disorder	Carbamazepine	tablet	100 mg	20
	Seizure Disorder	Carbamazepine	suspension	100 mg/5 ml	100 ml
9	Infectious disease	Cefazolin	injection	1 g, vial	1 vial
10	Infectious disease	Ceftriaxone	injection	500 mg vial	1 vial
11	Infectious disease	Chloramphenicol	tablet	250 mg	10
	Infectious disease	Chloramphenicol	injection	1 g, vial	1 vial
12	Infectious disease	Chlorpheniramine	tablet	4 mg	24
13	Infectious disease	Clarithromycin	suspension	125 mg/ 5 ml	100 ml
	Infectious disease	Clarithromycin	tablet	125 mg	10
14	Infectious disease	Cotrimoxazole	tablet	100 mg + 20 mg (also expressed as 400 mg + 80 mg)	10
	Infectious disease	Cotrimoxazole	suspension		100 ml
15	Seizure Disorder	Diazepam	tablet	5 mg	10
16	Anemia	Ferrous salt	suspension	30 mg/5 ml	100 ml
17	Fungal infection	Fluconazole	capsule	150 mg	6
18	Infectious disease	Gentamycin	injection	10 mg/ml	1 vial
19	Pain/inflammation	Ibuprofen	tablet	2 mg	24
	Pain/inflammation	Ibuprofen	suspension		100 ml
20	Tuberculosis	Isoniazide	tablet	100 mg	56
21	Pain	Morphine	tablet	10 mg	56
	Pain	Morphine	oral solution	10 mg/5 ml	100 ml
22	Dehydration	ORS	sachet	500 ml	1 sachet
23	Pain/inflammation	Paracetamol	tablet	250 mg	10
	Pain/inflammation	Paracetamol	suppository	125 mg	10
	Pain/inflammation	Paracetamol	suppository	250 mg	10
	Pain/inflammation	Paracetamol	suspension	125 mg	100 ml
24	Seizure Disorder	Phenobarbital	tablet	30 mg	10
	Seizure Disorder	Phenobarbital	injection	100 mg/ml	1 vial
25	Seizure Disorder	Phenytoin	tablet	50 mg,	90
	Seizure Disorder	Phenytoin	suspension	25 mg, 30 mg/5 ml	500 ml
26	Infectious disease	Procain penicillin	injection	1 g, vial	1 vial
27	Asthma	Salbutamol	inhaler	100mcg	1 inhaler (200 doses)
28	Xerophthalmia	Vitamin A	capsule	100.000 IU	50
29	Anemia	Vitamin B6	injection	50 mg/ml	1 vial
30	Dehydration	Zinc	tablet	20 mg	14
31	Spacer	Device			

The highest and lowest costs for each medicine were surveyed and brand or generic medicines were recorded. Medicines provided at public hospitals were procured by a general procurement (tender) process, therefore only the availability was recorded.

### Data collection

Trained data collectors visited pharmacy outlets and collected information regarding the cost, availability using a standardized data collection form and data were entered into a pre-programmed MS Excel Workbook provided as a part of the WHO/ HAI methodology. Data collectors provided the pharmacy outlets with written documentation describing the requirements for participation in the study. It was made clear that they could refuse to take part or terminate participation at any time. A verbal consent from the study participants was obtained prior to data collection.

### Availability of medicines

Availability of medicines was recorded on the day of data collection by the data collector and checked by the supervisor. Data collectors worked in pairs, visited selected medicine outlets, collected data and completed the Medicine Prices Data Collection Forms. A data entry person entered data from the hard copy Medicine Price Data Collection Forms into the electronic survey, and was responsible for data validation by running the double entry program to ensure accuracy. The Workbook’s auto checker was also used to assist in the verification process.

### Cross-country and international comparisons

Reference prices, developed by the Management Sciences for Health (MSH), were used to facilitate national and international comparisons as these were found to be the most useful standard [19].

Cost comparison was completed across the country and internationally, median prices were expressed as ratios relative to a standard set of international reference prices (Eq. 1):

1 $$ Median\ Price\ Ratio= median\ local\ unit\ price/ international\ reference\ unit\ price $$

The exchange rate used to calculate MPRs was 1 USD = 1988.5 Mongolian tugrigs as of the first day of data collection [20].

### Affordability

As defined by the WHO, affordability is estimated using the daily wage of the lowest-paid unskilled government worker by determining the number of days’ wages required to purchase selected courses of treatment for various conditions [18]. For the study, a total of seven conditions causing high pediatric mortality and morbidity in Mongolia and commonly used medicines were selected. These were; respiratory infections, urinary tract infections, otitis media, infections due to susceptible organisms, pneumonia, asthma, dehydration, pain and inflammation, seizure disorder and xerophthalmia. Treatment duration was defined as a full course of therapy for acute conditions whereas a supply of 30 days treatment duration was considered for chronic diseases [21].

## Results

### Availability of medicines on the day of data collection

**Fig. 1:**
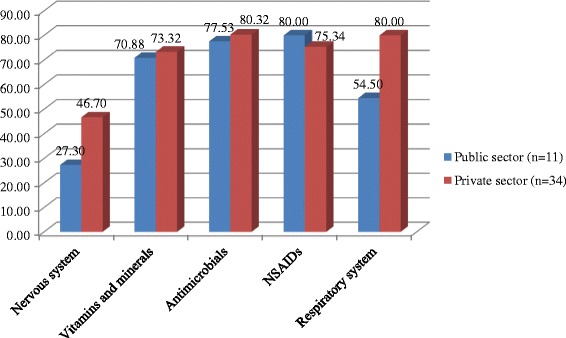
Average availability of the lowest cost medicines in the public and private sectors

There was a variation in availability of specific drugs however, a similar result was observed between the public and private sectors. The average availability of medicines was 72.6% in the public sector (range (9.1–100%). Correspondingly, the average availability of all selected medicines in the private sector was 76.7% (range 26.7–100%) (Fig. 1).

### Public and private sector patient coats

The lowest cost medicines were investigated and they were 1.95 times higher than the international reference price in the public sector. Generally, the lowest cost medicines were sold at 2.45 times higher than the international reference cost in the private sector. About one third of the medicines were costed at 1.85 (25 percentile) to 2.52 (75 percentile) times the international reference cost in the private sector with moderate cost variations existing across the sector (Table 2).

**Table 2:** Public and private sector patient prices

Medicine	Formulation	Target size pack	Lowest price medicines MPR (25th -75th percentile)	
			Public sector	Private sector
Albendazole	Tablet, 200 mg	2	3.83 (3.83–5.74)	3.83 (3.83–31.91)
Amoxicillin	Capsule, 250 mg	10	0.18 (0.17–0.34)	0.17(0.17–0.18)
Amoxicillin	Suspension, 125 mg	100 ml	2.51 (2.51–3.56)	4.19 (2.51–4.47)
Aminophyllin	Injection, 25 mg/ml	1 vial	–	–
Amoxicillin_Clavulanic acid	Tablet, 125 mg + 125 mg, tab	10	3.18 (3.01–3.26)	5.65 (3.51–5.65)
Amoxicillin_Clavulanic acid	Suspension, 125 mg + 31.25 mg	100 ml	2.03 (1.85–2.40)	4.59 (1.85–4.59)
Amoxicillin_Clavulanic acid	Suspension, 250 mg + 62.50 mg	100 ml	3.75 (3.28–4.37)	3.28 (3.16–3.30)
Azithromycin	Capsule, 250 mg	6	1.81 (1.63–1.90)	1.41 (0.80–1.86)
Azithromycin	Suspension, 200 mg/5 ml	100 ml	2.84 (2.63–2.84)	3.13 (2.84–3.13)
Beclomethason	Inhaler, 50 mg/ day	1 inhaler (200 doses)	–	–
Benzylpenicillin	Injection, 600 mg = 1 million IU	1 vial	0.89 (0.82–0.93)	1.41 (0.80–1.90)
Carbamazepin	Tablet, 100 mg	20	2.26 (2.03–3.59)	3.61 (2.03–3.61)
Carbamazepin	Suspension, 100 mg/5 ml	100 ml		
Cefazolin	Injection, 1 g, vial	1 vial	1.37 (1.31–1.54)	1.31 (1.31–1.43)
Ceftriaxone	Injection, 500 mg vial	1 vial	1.14 (1.05–1.31)	1.32 (1.32–1.58)
Chloramphenicol	Tablet, 250 mg	10	2.10 (1.99–2.21)	2.10 (1.99–2.21)
Chloramphenicol	Injection,1 g, vial	1 vial		
Chlorpheniramine	Tablet, 4 mg	24	17.01 (17.01–17.75)	17.38 (17.01–19.23)
Clarithromycin	Suspension, 125 mg/ 5 ml	100 ml	1.14 (1.05–1.33)	1.33 (1.33–1.39)
Clarithromycin	Tablet, 125 mg	10	1.51 (1.34–2.51)	2.35 (2.22–2.51)
Cotrimexazol	Tablet, 100 mg + 20 mg or 400 mg + 80 mg	10	3.56 (3.56–3.86)	3.96 (3.56–11.48)
Cotrimexazol	Suspension, 40 mg/5 ml	100 ml	0.41 (0.38–0.42)	0.42 (0.38–0.42)
Diazepam	Tablet, 5 mg	10	2.25 (2.14–3.05)	3.21 (3.10–4.28)
Ferrous salt	Suspension, 30 mg/5 ml	100 ml	0.53 (0.51–0.55)	0.65 (0.59–0.69)
Fluconazole	Capsule, 150 mg	6	0.70 (0.70–0.73)	3.02 (0.70–3.91)
Gentamycin	Injection, 10 mg/ml	1 vial	0.59 (0.59–0.64)	0.59 (0.59–0.80)
Ibuprofen	Tablet, 2 mg	24	12.38 (11.73–12.38)	12.38 (10.42–14.66)
Ibuprofen	Suspension, 100 mg/5 ml	100 ml	3.75 (3.76–4.46)	4.46 (4.46–4.64)
Isoniazide	Tablet, 100 mg	56	–	–
Morphine	Tablet, 10 mg	56	–	–
Morphine	oral solution, 10 mg/5 ml	100 ml	–	–
ORS	Sachet, 500 ml	1 sachet	4.02 (2.51–5.03)	4.53 (4.15–5.53)
Paracetamol	Tablet, 250 mg	10	–	–
Paracetamol	Suppository, 125 mg	10	1.32 (1.32–1.43)	1.32 (1.32–1.43)
Paracetamol	Suppository, 250 mg	10	1.42 (1.42–1.53)	1.48 (1.42–1.53)
Paracetamol	Suspension, 125 mg/5 ml	100 ml	3.17 (3.07–6.40)	7.92 (3.26–7.92)
Phenobarbital	Tablet, 30 mg		–	–
Phenobarbital	Injection, 100 mg/ml	1 vial	–	–
Phenytoin	Tablet, 50 mg	90	–	–
Phenytoin	Suspension, 25 mg,30 mg/5 ml	500 ml	–	–
Procain penicillin	Injection, 1 g, vial	1 vial	0.06 (0.06–0.07)	0.08 (0.06–0.11)
Salbutamol	Inhaler, 100mcg	1 inhaler (200 doses)	2.26 (1.45–2.51)	2.51 (2.49–2.58)
Vitamin A	Capsule, 100.000 IU	50	1.60 (1.60–2.26)	2.45 (1.60–2.52)
Vitamin B6	Injection, 50 mg/ml	1 vial	0.14 (0.14–0.17)	0.17 (0.17–0.23)
Zinc	Tablet, 20 mg	14	1.95 (1.67–2.23)	2.08 (1.67–2.23)

### Affordability of standard treatment regimens

Affordability of standard treatment regimens recommended by the WHO were analyzed for selected pediatric medicines. Lowest cost medicines in the private sector were less affordable than in the public sector for most conditions. As indicated in Table 3, lowest cost medicines in the public sector were more affordable for all conditions, with standard treatment costing a day’s wage or more. The least affordable treatment was estimated to be for respiratory tract infections or otitis media requiring amoxicillin clavulanic acid, suspension costing up to 1.03 days wages (Table 3).

**Table 3:** Affordability: Number of days’ wages of the lowest paid government worker needed to purchase standard medications

Disease condition and ‘standard’ treatment			Day’s wages to pay for treatment	
Condition	Drug name, strength, dosage form	Treatment schedule	LPM-Public sector	LPM-Private sector
Respiratory Tract Infections, UTIs	Amoxicllin Suspension 125 mg/5 ml	Child up to 10 years: 125 mg(=5 ml)x3x7 days = 105 ml	0.38	0.65
Respiratory Tract Infections, Otitis Media	Amoxicillin Clavulanic Acid Suspension 125–31.25 mg/5 ml	Child 1–6 years: 125 mg(=5 ml)x3x7 days = 105 ml	0.46	1.03
Respiratory Tract Infections, Otitis Media	Amoxicillin Clavulanic Acid Suspension 250–62.5 mg/5 ml	Child over 10 years: 250 mg(=5 ml)x3x7 days = 105 ml	0.85	1.00
Seizure Disorder	Carbamazepine tablet, 100 mg	Maintenance treatment: 5 mg/kg*18 kg*3*30 days = 8100 mg or 40.5 tablets for 1 month	0.34	0.54
Infections due to Susceptible Organisms	Ceftriaxone 500 mg vial	Child under 50 kg: Maximum 1 g dailyx7 days = 7 g or 14 vials	0.11	0.15
Infections due to Susceptible Organisms	Sulfamethoxazole+Trimethoprim (400 mg + 80 mg)	18kgx4 mg/kg = 72 mg TMPx2x7days = 1008 mg.126 ml or 12.6 tablets total for 7 days	0.09	0.30
Dehydration	Oral Rehydration Solution, powder to make 500 ml	Moderate Dehydration; 75 ml/kgx18kg = 1350 ml	0.06	0.12
Pneumonia	Amoxicllin Suspension 125 mg/5 ml	Child up to 10 years: 125 mg(=5 ml)x3x7 days = 105 ml	0.38	0.65
Pain/inflammation	Paracetamol Suspension 24 mg/ml	5 year old child: 15 mg/kgx18kgx4x3 = 3240 mg = (130 mL)	0.28	0.28
Pain/inflammation	Ibuprofen suspension	5 year old child: 15 mg/kgx18kgx4x3 = 3240 mg = (130 mL)	0.67	0.79
Asthma	Salbutamol inhaler 100 mcg/dose	1 inhaler 200 doses	0.38	0.65
Xerophthalmia	Vitamin A 50,000 units	Child 1–12 years: 200,000 units × 3 doses	0.12	0.18

*LPM- Lowest Price Medicine

## Discussion

The study has evaluated the availability and affordability of essential pediatric medicines in Mongolia. The average availability of essential pediatric medicines was relatively good being on average over 70% in both sectors. Inconsistent and higher costs and lower affordability of medicines was found in the private sector. Pediatric formulations recommended in the WHO EMLc, including beclomethasone- inhaler [1], carbamazepine- suspension [1], diazepam-rectal solution [1], chloramphenicol – injection [1], isoniazid- scored tablet [1], morphine-oral solution [1], phenobarbital -injection [1], phenytoin- chewable tablet [1] spacer- device [1] were not assessable, because they are not registered and unavailable in Mongolia. Similarly, lack of appropriate dosage formulations for phenobarbital, phenytoin, carbamazepine, diazepam (rectal solution) were reported in a study at a tertiary care hospital with 2000 beds, in Western India [22].

This identifies a lack of some child friendly formulations available which could be related to the difficulty of obtaining the product if it was not locally manufactured. A study has assessed the availability of essential pediatric medicines in China and reported that a dearth of registered pediatric medicines may be attributed to multiple factors including the manufacturers’ lack of motivation to produce because of low demand or small profit, less effective bidding and procurement systems for pediatric medicines, hospitals’ preferences for higher-priced medicines, inappropriate drug selection and weak health and supply systems [15, 23]. Additionally, insufficient knowledge or application of clinical standards and protocols [24], and exclusion from central procurement lists due to high cost may be other reasons for unavailability [25].

Amongst the surveyed medicines, originator brands were rarely available in both sectors, mainly due to high cost and the Government’s effort to improve and promote the use of generic medicines in Mongolia [5]. The study results were consistent with previous studies assessing availability and affordability of essential medicines for adults [13, 16, 26–28]. These studies have shown that availability was lower in the public sector when compared to the private pharmacies.

Lack of child friendly formulations was observed in this study. Chloramphenicol injection, morphine, beclomethasone were not available in two sectors. According to other studies, similar findings have been reported [23, 29]. Medicines to treat seizure disorders (phenobarbital, phenytoin) and pain (morphine) were not found in any of the surveyed pharmacy outlets. This is due to the legislation restricting the use of narcotic and psychotropic drugs [4], only a few pharmacy outlets with special permission are eligible to stock them.

Diseases of the digestive system, including diarrhea was one of the main reasons for pediatric mortality and morbidity in Mongolia. Oral rehydration powders were available in most of pharmacy outlets in both sectors. However, dispersible tablets of Zinc as recommended in the EMLc, were available in only half of the surveyed sites (50.6%). Evidence based data suggest that zinc is beneficial for treatment of pediatric diarrhea for children aged 6 months or older [30].

Paracetamol (acetaminophen) has a unique role in children because it is the first-line choice for the treatment of both fever and pain. When used in the recommended doses, it has few side effects and is remarkably well tolerated [31]. However, the recommended pediatric dose tablet was not available in any of the surveyed pharmacies. Instead, dispensers (pharmacist or pharmacy technician) would dispense adult dose tablet (500 mg) and advise to cut the tablet into half when a pediatric dose was prescribed. Clinical data proves that in overdose, paracetamol is hepatotoxic [31].

The affordability of lowest cost medicines was compared across the country. The pediatric essential medicines were generally less affordable in the private sector and costed more than the recommended international reference prices.

Mongolia is country with seasonal variations, with winter being very long and harsh. During this time, most pediatric conditions are associated with respiratory infections. Pneumonia and other respiratory infections are reported to be the main reason for children’s hospitalization in Mongolia [3]. Empiric treatment with appropriate antibiotics for respiratory infections, including pneumonia is crucial in the reduction of mortality from pneumonia [32]. Treatment of pneumonia with amoxicillin suspension would cost more than a half day’s wage, whereas for other respiratory infections (otitis media), the cost of treatment would be 1 day’s wage.

When compared to other countries, this result is comparably low [29]. Considering the fact that 14% of Mongolians live on less than USD 1 per day [2], treatments seem to be too expensive. On the other hand, treatment cost reflects medicines cost only, excluding any additional consultation or diagnostic costs. Additionally, treatment was estimated only for one child, indicating that families with more children and their treatment would endure an overwhelming cost. Similar to previous findings, essential medicines are often not affordable for many populations [13, 16, 27, 33].

### Limitations

The study has several limitations that are consistent with previous surveys. The WHO/HAI methodology did not assess the therapeutic alternatives or alternative dosage forms. The study results reflect the status of availability and cost based on the day of data collection. They do not necessarily reflect the monthly or yearly availability of essential pediatric medicines at the national level or individual pharmacy outlets. In addition, the median cost ratio was estimated using the supplier cost. Although, when the supplier cost was not available, buyer costs were used to calculate the median international reference cost. Substituting the supplier with buyer cost could result in inaccurate prices and therefore influence the true median price [15].

On the other hand, the study has utilized a previously validated methodology using a standardized way to evaluate the availability and costs of essential pediatric medicines in the country [34]. Furthermore, training and using multiple checkpoints have improved the quality of data collection, data entry and interpretation.

## Conclusion

The findings of this study can serve as basic data to develop and revise the National Policy in order to improve the accessibility and availability of essential pediatric medicines in Mongolia. Furthermore, control, monitoring and comparing pediatric medicines costs not just across the country but also at the international level would help to increase transparency of different tasks including registration, procurement and reimbursement decision making procedures. A detailed investigation regarding the prescribing of pediatric medicines should be completed to identify the challenges and barriers. Finally, the procurement, supply and distribution of essential pediatric medicines needs to be examined in order to identify the lack of availability and higher costs of some essential pediatric medicines in Mongolia.

## Abbreviations

DRF: Drug Revolving Fund
EMLc: The Essential Medicines List for Children
GDP: Gross Domestic Product
HAI: Health Action International
IRP: International Reference Price
MDG: Millennium Development Goals
NDPM: National Drug Policy of Mongolia
NEDL: National Essential Drug List
NSAID: Non steroidal anti-inflammatory drugs
WHO: World Health Organization
